# Association of Cystatin C Kidney Function Measures With Long-term Deficit-Accumulation Frailty Trajectories and Physical Function Decline

**DOI:** 10.1001/jamanetworkopen.2022.34208

**Published:** 2022-09-30

**Authors:** Chenglong Li, Yanjun Ma, Chao Yang, Rong Hua, Wuxiang Xie, Luxia Zhang

**Affiliations:** 1 Peking University Clinical Research Institute, Peking University First Hospital, Beijing, China; 2 Heart and Vascular Health Research Center, Peking University Clinical Research Institute, Peking University Shougang Hospital, Beijing, China; 3 Key Laboratory of Molecular Cardiovascular Sciences, Peking University, Ministry of Education, Beijing, China; 4 National Institute of Health Data Science, Peking University, Beijing, China; 5 Advanced Institute of Information Technology, Peking University, Hangzhou, China; 6 Renal Division, Department of Medicine, Peking University First Hospital, Peking University Institute of Nephrology, Beijing, China

## Abstract

**Importance:**

It remains unclear whether cystatin C and cystatin C–based kidney function measures are associated with frailty trajectories and physical function decline.

**Objective:**

To examine the associations of cystatin C level, cystatin C estimated glomerular filtration rate (eGFRcys), and the difference between eGFRs (eGFRdiff) using cystatin C and creatinine levels with long-term deficit-accumulation frailty trajectories and physical function decline.

**Design, Setting, and Participants:**

This prospective cohort study used data from 15 949 participants in the China Health and Retirement Longitudinal Study (CHARLS) and the US Health and Retirement Study (HRS), 2 ongoing nationally representative cohort studies enrolling community-dwelling older people. Biennial surveys, known as waves, are conducted in both the CHARLS and the HRS. Seven-year data from wave 1 (May 2011 to March 2012) to wave 4 (July to September 2018) in the CHARLS and 12-year data from wave 8 (March 2006 to February 2007) to wave 14 (April 2018 to June 2019) in the HRS were assessed, with wave 1 in the CHARLS and wave 8 in the HRS serving as baseline waves. Data were analyzed from February 12 to May 20, 2022.

**Exposures:**

Baseline serum cystatin C and creatinine levels. Cystatin C eGFR and creatinine estimated GFR (eGFRcr) were calculated using the 2021 race-free equations developed by the Chronic Kidney Disease Epidemiology Collaboration. The difference between eGFRcys and eGFRcr was calculated by subtracting eGFRcr from eGFRcys.

**Main Outcomes and Measures:**

Based on 12-year follow-up data from the HRS and 7-year follow-up data from the CHARLS, a 29-item deficit-accumulation frailty index (FI) was constructed to assess frailty trajectories at each visit. Physical function decline was evaluated using repeated objective physical function measurements (grip strength and gait speed). Linear mixed models were used to examine longitudinal associations.

**Results:**

Among 15 949 older adults included in the analysis, 9114 participants were from the HRS (mean [SD] age, 66.2 [10.1] years; 5244 women [57.5%]), and 6835 were from the CHARLS (mean [SD] age, 58.4 [9.8] years; 3477 women [50.9%]). With regard to race and ethnicity, the HRS cohort included 7755 White individuals (85.1%) and 1359 individuals (14.9%) of other races and/or ethnicities (including American Indian or Alaska Native, Asian, Black or African American, Native Hawaiian or Pacific Islander, and other); all participants in the CHARLS cohort were of Chinese ethnicity. Each SD increment in serum cystatin C was associated with a faster increase in FI in both the HRS cohort (β = 0.050 SD/y; 95% CI, 0.045-0.055 SD/y; *P* = .001) and the CHARLS cohort (β = 0.051 SD/y; 95% CI, 0.042-0.060 SD/y; *P* = .001). An inverse association was observed for eGFRCys (HRS cohort: β = −0.058 SD/y; 95% CI, −0.062 to −0.053 SD/y; *P* = .001; CHARLS cohort: β = −0.056 SD/y; 95% CI, −0.064 to −0.047 SD/y; *P* = .001). These associations remained after controlling for serum creatinine (β = 0.051 SD/y; 95% CI, 0.042-0.060 SD/y; *P* = .001) and eGFRcr (β = −0.056 SD/y; 95% CI, −0.064 to −0.047 SD/y; *P* = .001) in the CHARLS cohort. Similar to the results observed for eGFRcys, each SD increment in the eGFRdiff was associated with a slower increase in FI (β = −0.027 SD/y; 95% CI, −0.035 to −0.018 SD/y; *P* = .001) in the CHARLS cohort. Similar findings were observed for physical function decline. For example, each SD increment in serum cystatin C was associated with faster decreases in both grip strength (β = −0.006 SD/y; 95% CI, −0.008 to −0.003 SD/y; *P* = .001) and gait speed (β = −0.007 SD/y; 95% CI, −0.011 to −0.003 SD/y; *P* = .001) in the HRS cohort and faster decreases in gait speed (β = −0.017 SD/y; 95% CI, −0.027 to −0.006 SD/y; *P* = .002) in the CHARLS cohort.

**Conclusions and Relevance:**

In this cohort study, cystatin C, eGFRcys, and eGFRdiff were associated with long-term frailty trajectories and physical function decline among community-dwelling older people without frailty. Monitoring kidney function using cystatin C could have clinical utility in identifying the risk of accelerated frailty progression.

## Key Points

**Question:**

Are kidney function measures, including cystatin C level, cystatin C estimated glomerular filtration rate (eGFRcys), and the difference between eGFRs (eGFRdiff) using cystatin C and creatinine, associated with long-term deficit-accumulation frailty trajectories and physical function decline?

**Findings:**

In this cohort study of 15 949 participants enrolled in 2 population-based cohorts with nationally representative samples in the US and China, cystatin C level, eGFRcys, and eGFRdiff were consistently and independently associated with accelerated deficit-accumulation frailty trajectories and faster decline rates in all objective physical function measurements, even after controlling for creatinine level and creatinine eGFR.

**Meaning:**

This study’s findings suggest that, among community-dwelling older people without frailty, monitoring kidney function using cystatin C could provide additional insight into identifying the risk of accelerated frailty progression and physical function decline.

## Teaser

This cohort study uses data from 2 nationally representative cohorts in China and the US to assess the associations of cystatin C kidney function measures with long-term deficit-accumulation frailty trajectories and physical function decline among older adults without frailty.

## Introduction

Frailty is emerging as a substantial health challenge as the world's aging population grows and has been associated with adverse outcomes, including mortality, hospitalization, and falls.^1,2,3,4^ There are currently 2 operational definitions for frailty; the first defines frailty as the accumulation of deficits, and the second defines frailty as a physical phenotype.^5^ Despite these heterogeneous definitions, frailty is deemed a dynamic process that worsens with aging.^1^ Therefore, evaluating long-term frailty trajectories (eg, the longitudinal patterns of increasing frailty over time) and identifying potentially modifiable risk factors could be important for developing effective strategies of prevention.^6^

Cystatin C has emerged as a novel indicator for kidney function, with the ability to eliminate the negative consequences of traditional race-based approaches.^7^ Compared with creatinine estimated glomerular filtration rate (eGFRcr), cystatin C estimated GFR (eGFRcys) could offer a measure that is less dependent on muscle mass.^8^ Cystatin C eGFR has also been found to be associated with frailty.^9,10,11^ However, previous studies^9,10,11^ have concentrated on dichotomized frailty status at a single time point without taking into account dynamic frailty trajectories. In addition, the difference between eGFRcys and eGFRcr, namely, eGFRdiff, also has clinical implications among the aging population, and uncertainty remains regarding the prospective association of eGFRdiff with long-term frailty trajectories.^10^ Serum creatinine levels are more likely to be altered by muscle mass than by cystatin C levels, and higher eGFRcr (ie, lower creatinine) may indicate low muscle mass.^8^ Therefore, the negative eGFRdiff may be explained in part by sarcopenia, which is associated with a variety of negative outcomes in older adults.^12^

We intended to investigate the prospective association of kidney function measures, including serum cystatin C, eGFRcys, and eGFRdiff, with long-term frailty trajectories. Given that decline in physical function is an important phenotype of frailty progression, we incorporated it into the evaluation.^5^ Data were obtained from the Health and Retirement Study (HRS) and the China Health and Retirement Longitudinal Study (CHARLS), 2 population-based cohort studies with nationally representative samples from the US and China. We hypothesized that serum cystatin C, eGFRcys, and eGFRdiff would be associated with long-term accelerated frailty trajectories and physical function decline.

## Methods

### Study Population

The HRS and CHARLS are 2 ongoing prospective and nationally representative cohorts of community-dwelling adults (aged ≥50 years in the HRS and ≥45 years in the CHARLS). The baseline survey in the HRS was conducted in the US from April 1992 to March 1993, and the latest released follow-up survey was conducted from March 2020 to May 2021. The baseline survey in the CHARLS was conducted in China from May 2011 to March 2012, and the latest released follow-up survey was conducted from July to September 2018. Details about the 2 cohorts’ objectives, design, and methods can be found elsewhere.^13,14^ The HRS was approved by the institutional review boards of the University of Michigan and the National Institute on Aging, and the CHARLS was approved by the institutional review board of Peking University. Before being included in the studies, all participants provided written informed consent. The current study followed the Strengthening the Reporting of Observational Studies in Epidemiology (STROBE) reporting guideline.

**Figure 1.**
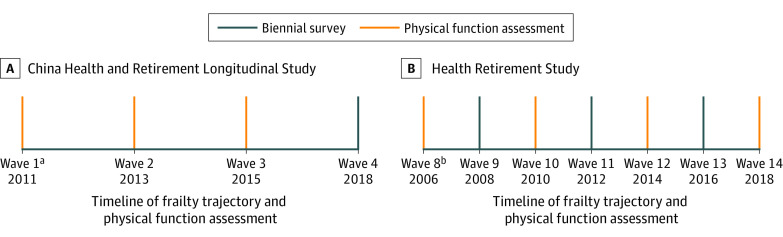
Study Timeline and Design ^a^During wave 1, baseline cystatin C and creatinine levels were assessed. ^b^During wave 8, only baseline cystatin C level was assessed.

Biennial surveys, known as waves, were conducted in both the CHARLS and the HRS. We used 7-year data from wave 1 (May 2011 to March 2012) to wave 4 (July to September 2018) in the CHARLS and 12-year data from wave 8 (March 2006 to February 2007) to wave 14 (April 2018 to June 2019) in the HRS, with wave 1 in the CHARLS and wave 8 in the HRS serving as baseline waves. Data were analyzed from February 12 to May 20, 2022. The study timeline and design are shown in Figure 1. Participants were excluded if they were missing serum cystatin C values at baseline, had frailty at baseline, or were unavailable for follow-up. A total of 20 228 participants (9355 from the HRS cohort and 10 873 from the CHARLS cohort) were excluded from the analysis, and 15 949 participants (9114 from the HRS cohort and 6835 from the CHARLS cohort) were included.

### Kidney Function

Both cohort studies conducted kidney function measurements at a central laboratory, with standardized protocol applied. At baseline, the HRS obtained dried blood samples to measure serum cystatin C level, and the CHARLS collected venous blood samples to measure cystatin C and creatinine levels. The HRS team further performed adjustments to measured values and derived measurements equivalent to the Third National Health and Nutrition Examination Survey.^15^ This approach was used to make the level of the HRS data more similar to the level based on conventional venous blood assays.^16^

We used 2021 race-free equations developed by the Chronic Kidney Disease Epidemiology Collaboration^17^ to calculate eGFRs using baseline serum cystatin C values (from the CHARLS and HRS), baseline serum creatinine values (from the CHARLS only), sex, and age. The calculated eGFRs were categorized into 3 groups: (1) less than 60 mL/min/1.73 m^2^, (2) 60 to 89 mL/min/1.73 m^2^, and (3) 90 mL/min/1.73 m^2^ or greater.^18^ We calculated the difference between the 2 eGFRs as eGFRcys − eGFRcr, denoted as eGFRdiff (CHARLS only). To assess whether the eGFRdiff captured eGFRcys relative to eGFRcr, we also adjusted for eGFRcr in the analysis regarding eGFRdiff.

### Frailty Evaluation

We followed a standard procedure to construct a frailty index (FI), based on the deficit accumulation model developed by Searle et al.^19^ To construct the FI, we selected 29 items, including functional limitations, self-reported health status, depressive symptoms, medical conditions, and cognition status. The 29 items included in the FI were identical across different waves (details of item definitions are shown in the eMethods and eTable 1 in the Supplement). Frailty status was defined as a value of 0.25 or greater on the FI.^4^

### Physical Function

Both the HRS and the CHARLS measured physical function at regular intervals, with standardized measurement protocols implemented by trained research nurses. Measurements included grip strength and timed walk. For the HRS, all measurements were conducted at wave 8 (2006-2007), wave 10 (2010-2011), wave 12 (2014-2015), and wave 14 (2018-2019). For the CHARLS, all measurements were conducted consecutively from wave 1 (2011) to wave 3 (2015). The administered measurements had been verified with good validity in older adults.^20^

### Covariates

Covariates included both sociodemographic and clinical characteristics assessed at baseline. Sociodemographic characteristics included age (in years), sex, race and ethnicity (White vs other races and/or ethnicities), living arrangements (living alone vs not living alone), socioeconomic status factors (annual family income, educational level, employment status, and medical insurance coverage), current smoking (yes vs no), alcohol consumption (≥3 days/wk vs <3 days/wk), and physical activity (moderate or vigorous activity no less than once per week vs moderate or vigorous activity less than once per week). Clinical characteristics included overweight status, physical disability, and comorbidities, including hypertension, diabetes, stroke, heart diseases, chronic lung diseases, and cancer. Overweight was defined as having a body mass index (calculated as weight in kilograms divided by height in meters squared) of 25 or higher for the HRS cohort and 24 or higher for the CHARLS cohort.^21^ Physical disability was defined as difficulty in performing 1 or more of the following basic activities of daily living: bathing, dressing, eating, getting in and out of bed, and walking across a room. Hypertension was defined as a physician-confirmed diagnosis, mean systolic/diastolic blood pressure of 140/90 mm Hg or higher, or use of antihypertensive medication. Diabetes was defined as a physician-confirmed diagnosis, a fasting plasma glucose level of 126 mg/dL or higher (to convert to mmol/L, multiply by 0.0555 [ie, ≥7.0 mmol/L]), a glycated hemoglobin A_1c_ level of 6.5% (to convert to mmol/mol, multiply by 10.93 and subtract 23.5 [ie, ≥48 mmol/mol]), or receipt of insulin injections. Stroke, heart diseases, chronic lung diseases, and cancer were defined as physician-confirmed formal diagnoses reported by participants.

We considered 4 socioeconomic factors, including annual family income, educational level, employment status, and medical insurance coverage. Annual family income was assessed using sample tertiles of total income at the household level for the last calendar year. In the HRS cohort, tertile 1 indicated $0 to $24 000; tertile 2, $24 001 to $55 916; and tertile 3, $55 932 to $25 360 120. In the CHARLS cohort, tertile 1 indicated −$85 219 to $952; tertile 2, $954 to $4778; and tertile 3, $4781 to $406 095 (to convert to Chinese yuan, multiply by 6.3 [all conversions were rounded]; the reason for the negative value in tertile 1 was consideration of the cost of energy, housing or equipment rental, raw materials, transportation, marketing, wages, taxes, and other fees). Education was categorized into 3 levels, including less than high school, high school or equivalent (general educational development), and college or higher. Employment status was grouped as employed (including participants with paid employment, participants who were self-employed, and participants who were retired) and unemployed. Medical insurance coverage was categorized into 3 types: private health insurance plans, public health insurance plans, and uninsured.

### Statistical Analysis

Means with SDs or medians with IQRs were used for reporting descriptive statistics of continuous variables, and numbers with percentages were used for reporting categorical variables. Differences in characteristics were assessed using the *t* test, Wilcoxon rank sum test, or χ^2^ test. After assessing the heterogeneity between the 2 cohorts, we evaluated the appropriateness of performing a pooled analysis.

Based on multiple repeated measurements (including baseline values) of the FI and physical function in individual participants, we used linear mixed models to assess longitudinal associations between baseline kidney function measures and the annual rate of change in FI and physical function during follow-up. Such models have been widely used for handling multiple repeated measurements of continuous outcomes and can incorporate all available follow-up data to derive the rate of change in outcomes.^22,23,24^ We included the following terms in the models: kidney function measures, time (years from baseline to the end of follow-up), the interaction between kidney function measures and time, and covariates. The slope of the time represented the annual rate of change in the outcome, and the interaction between kidney function measures and time represented the association between kidney function measures and the annual rate of change in the outcome. We also fitted the intercept and slope of the time variable as random effects at the participant level to account for between-participant differences in the outcome at baseline and the rate of change during follow-up, with the Toeplitz covariance structure^25^ used for modeling within-participant correlations between repeated outcome measurements. Considering that the linear mixed models could appropriately handle dependent variable observations that were missing at random, no further imputation procedures were applied.^26^ For all continuous outcome and kidney function measures, baseline means and SDs were used to conduct the *z* score standardization.

We further applied an inverse probability weighting approach to handle potential selection bias. The included study samples were reweighted, and the analytical weight for each individual was calculated as the inverse of the probability of being included in the analysis.^27^ We used binary logistic regression analysis to estimate the weights, with covariates identical to those used in the primary analysis included in the model. Absolute standardized mean differences were used to assess the differences between included and excluded participants, with a Love plot^28^ selected for visualization.

Several sensitivity analyses were conducted. First, to evaluate potential selection bias, we conducted a nonresponse analysis by comparing baseline characteristics of included and excluded participants. Second, in addition to analyzing frailty trajectories using linear mixed models, we used a group-based trajectory modeling approach to identify potential frailty trajectories, and we examined associations between kidney function measures and frailty trajectories using logistic regression analysis (with details described in eMethods in the Supplement). This part of the analysis was performed to evaluate the sensitivity of our main findings to different longitudinal data modeling techniques. We conducted our primary analysis based on the original unweighted samples to further examine the robustness of our main findings.

Statistical analysis was conducted using SAS software, version 9.4 (SAS Institute Inc), and R software, version 3.6.2 (R Foundation for Statistical Computing). Two-tailed *P* = .05 was considered statistically significant.

## Results

### Study Population

Among 15 949 individuals included in the analysis, 9114 participants (mean [SD] age, 66.2 [10.1] years; 3870 men [42.5%] and 5244 women [57.5%]; median follow-up, 12 years [IQR, 8-12 years]) were from the HRS, and 6835 participants (mean [SD] age, 58.4 [9.8] years; 3358 men [49.1%] and 3477 women [50.9%]; median follow-up, 7 years [IQR, 7-7 years]) were from the CHARLS. The detailed inclusion process is shown in eFigure 1 in the Supplement. With regard to race and ethnicity, the HRS cohort included 7755 White individuals (85.1%) and 1359 individuals (14.9%) of other races and/or ethnicities (including American Indian or Alaska Native, Asian, Black or African American, Native Hawaiian or Pacific Islander, and other); all participants in the CHARLS cohort were of Chinese ethnicity.

**Figure 2.**
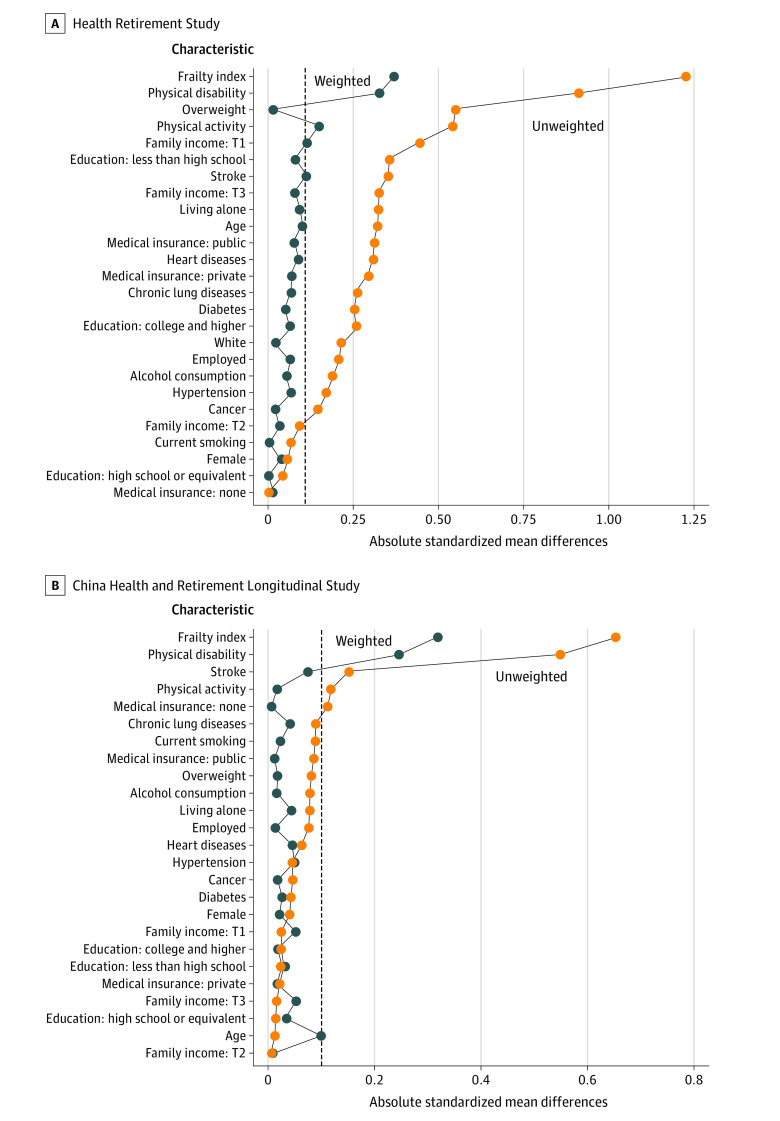
Differences in Characteristics Between Included and Excluded Participants in 2 Independent Cohorts Love plots show differences based on the original unweighted and inverse probability–weighted samples. In the Health Retirement Study (HRS) cohort, family income tertile 1 (T1) indicates $0 to $24 000; tertile 2 (T2), $24 001 to $55 916; and tertile 3 (T3), $55 932 to $25 360 120. In the China Health and Retirement Longitudinal Study (CHARLS) cohort, family income tertile 1 indicates −$85 219 to $952; tertile 2, $954 to $4778; and tertile 3, $4781 to $406 095 (to convert to Chinese yuan, multiply by 6.3).

Differences in most baseline characteristics were observed between the HRS cohort vs the CHARLS cohorts (Table 1). Hence, no formal pooled analysis was conducted, and results were reported within each cohort instead. After inverse probability weighting, the differences between included and excluded participants were smaller compared with the differences between the original unweighted samples (Figure 2). The distribution of outcome measurements at different waves is shown in eTable 2 and eTable 3 in the Supplement.

**Table 1.** Baseline Characteristics of Participants in 2 Independent Cohort Studies

Characteristics	Participants, No. (%)		*P* value^a^
	CHARLS (n = 6835)	HRS (n = 9114)	
Sex			
Male	3358 (49.1)	3870 (42.5)	.001
Female	3477 (50.9)	5244 (57.5)	
Age, mean (SD), y	58.4 (9.8)	66.2 (10.1)	.001
Follow-up duration, median (IQR), y	7 (7-7)	12 (8-12)	.001
Race			
Chinese	6835 (100)	NA	NA
White	0	7755 (85.1)	
Other^b^	0	1359 (14.9)	
Living alone	759 (11.1)	2458 (27.0)	.001
Educational level			
Less than high school	6036 (88.3)	1372 (15.1)	.001
High school or equivalent	676 (9.9)	3316 (36.4)	
College and higher	123 (1.8)	4426 (48.6)	
Annual family income tertile^c^			
1	2244 (32.8)	2085 (22.9)	.001
2	2288 (33.5)	3238 (35.5)	
3	2303 (33.7)	3791 (41.6)	
Employment status			
Unemployed	260 (3.8)	843 (9.2)	.001
Employed	6575 (96.2)	8271 (90.8)	
Medical insurance coverage			
Uninsured	435 (6.4)	507 (5.6)	.001
Public	6214 (90.9)	2237 (24.5)	
Private	186 (2.7)	6370 (69.9)	
Current smoking	2054 (30.1)	1141 (12.5)	.001
Alcohol consumption	1005 (14.7)	1834 (20.1)	.001
Physical activity	1924 (28.1)	7487 (82.1)	.001
Overweight	2750 (40.2)	6608 (72.5)	.001
Physical disability	283 (4.1)	193 (2.1)	.001
Serum cystatin C, mean (SD), mg/L	1.00 (0.24)	1.04 (0.38)	.001
eGFRcys, mean (SD), mL/min/1.73 m^2^	79.88 (20.69)	75.98 (23.87)	.001
Serum creatinine, mean (SD), mg/dL	0.79 (0.19)	NA	NA
eGFRcr, mean (SD), mL/min/1.73 m^2^	96.25 (14.36)	NA	NA
eGFRdiff, mean (SD), mL/min/1.73 m^2^	−16.39 (16.86)	NA	NA
Frailty index, median (IQR)	0.09 (0.05-0.16)	0.11 (0.05-0.16)	.001
Grip strength, mean (SD), kg	32.36 (10.45)	32.79 (11.03)	.02
Gait speed, mean (SD), cm/s	64.56 (21.01)	84.99 (23.87)	.001
Hypertension	2667 (39.0)	5682 (62.3)	.001
Diabetes	1020 (14.9)	1470 (16.1)	.04
Stroke	88 (1.3)	344 (3.8)	.001
Heart diseases	737 (10.8)	1572 (17.2)	.001
Chronic lung diseases	548 (8.0)	427 (4.7)	.001
Cancer	46 (0.7)	1040 (11.4)	.001

Abbreviations: CHARLS, China Health and Retirement Longitudinal Study; eGFRcr, estimated glomerular filtration rate using serum creatinine; eGFRcys, eGFR using serum cystatin C; eGFRdiff, difference between eGFRcys and eGFRcr (calculated as eGFRcys − eGFRcr); HRS, Health and Retirement Study; NA, not applicable.

SI conversion factor: To convert creatinine to micromoles per liter, multiply by 76.25.

^a^ *P* values for differences between groups were derived using a *t* test, χ^2^ test, or Wilcoxon rank sum test.

^b^ In the HRS cohort, participants with races and ethnicities other than White were included in the *other* group to protect their anonymity. These other races and ethnicities included American Indian or Alaska Native, Asian, Black or African American, Native Hawaiian or Pacific Islander, and other. In the CHARLS cohort, all participants were of Chinese ethnicity.

^c^ In the HRS cohort, tertile 1 indicated $0 to $24 000; tertile 2, $24 001 to $55 916; and tertile 3, $55 932 to $25 360 120. In the CHARLS cohort, tertile 1 indicated −$85 219 to $952; tertile 2, $954 to $4778; and tertile 3, $4781 to $406 095 (to convert to Chinese yuan, multiply by 6.3).

### Associations Between Kidney Function Measures and Frailty Trajectories

Each SD increment in serum cystatin C level was associated with a faster increase in FI in both the HRS cohort (β = 0.050 SD/y; 95% CI, 0.045-0.055 SD/y; *P* = .001) and the CHARLS cohort (β = 0.051 SD/y; 95% CI, 0.042-0.060 SD/y; *P* = .001) (Table 2). An inverse association for eGFRcys was observed in both the HRS cohort (β = −0.058 SD/y; 95% CI, −0.062 to −0.053 SD/y; *P* = .001) and the CHARLS cohort (β = −0.056 SD/y; 95% CI, −0.064 to −0.047 SD/y; *P* = .001). In the CHARLS cohort (which had information on serum creatinine levels), the associations of serum cystatin C (β = 0.051 SD/y; 95% CI, 0.042-0.060 SD/y; *P* = 01) and eGFRcys (β = −0.056 SD/y; 95% CI, −0.064 to −0.047 SD/y; *P* = .001) with frailty trajectories remained after further adjusting for serum creatinine and eGFRcr, respectively. Among participants in the CHARLS, each SD eGFRdiff increment was associated with a slower increase in FI (β = −0.027 SD/y; 95% CI, −0.035 to −0.018 SD/y; *P* = .001).

**Table 2.** Longitudinal Associations Between Kidney Function Measures and Frailty Trajectories in 2 Independent Cohorts Based on Inverse Probability–Weighted Samples

Kidney function measure	Rate of change in frailty index score, SD/y	
	β (95% CI)^a^	*P* value
**HRS (n = 9114)**		
eGFRcys, mL/min/1.73 m^2^		
<60	0.144 (0.131 to 0.157)	.001
60-89	0.044 (0.033 to 0.054)	.001
≥90	0 [Reference]	NA
Test for linear trend^b^	0.070 (0.064 to 0.076)	.001
Continuous serum cystatin C per SD^c^	0.050 (0.045 to 0.055)	.001
Continuous eGFRcys per SD^c^	−0.058 (−0.062 to −0.053)	.001
**CHARLS (n = 6835)**		
eGFRcys, mL/min/1.73 m^2^		
<60	0.167 (0.140 to 0.193)	.001
60-89	0.031 (0.012 to 0.050)	.001
≥90	0 [Reference]	NA
Test for linear trend^b^	0.073 (0.061 to 0.086)	.001
Continuous serum cystatin C per SD^c^	0.051 (0.042 to 0.060)	.001
Continuous eGFRcys per SD^c^	−0.056 (−0.064 to −0.047)	.001
Continuous serum creatinine per SD^c^	0.004 (−0.005 to 0.013)	.40
Continuous eGFRcr per SD^c^	−0.048 (−0.057 to −0.039)	.001
Continuous cystatin C adjusted for creatinine per SD^c^	0.051 (0.042 to 0.060)	.001
Continuous eGFRcys adjusted for eGFRcr per SD^c^	−0.056 (−0.064 to −0.047)	.001
eGRFdiff adjusted for eGFRcr per SD^c^	−0.027 (−0.035 to −0.018)	.001

Abbreviations: CHARLS, China Health and Retirement Longitudinal Study; eGFRcr, estimated glomerular filtration rate using serum creatinine; eGFRcys, eGFR using serum cystatin C; eGFRdiff, difference between eGFRcys and eGFRcr (calculated as eGFRcys − eGFRcr); HRS, Health and Retirement Study; NA, not applicable.

^a^ β Coefficient was estimated using linear mixed models, with positive value representing accelerated frailty. Adjusted covariates include age, sex, race and ethnicity, living alone, educational level, annual household income, employment status, health insurance status, current smoking, alcohol consumption, physical activity, overweight status, physical disability, hypertension, diabetes, stroke, heart diseases, chronic lung diseases, and cancer.

^b^ Performed by treating eGFRcys categories as a numerical variable.

^c^ Estimated as the β coefficient for 1 SD increment in kidney measures.

### Associations Between Kidney Function Measures and Physical Function Decline

Each SD increment in serum cystatin C level was associated with faster decreases in both grip strength (β = −0.006 SD/y; 95% CI, −0.008 to −0.003 SD/y; *P* = .001) and gait speed (β = −0.007 SD/y; 95% CI, −0.011 to −0.003 SD/y; *P* = .001) in the HRS cohort and faster decreases in gait speed (β = −0.017 SD/y; 95% CI, −0.027 to −0.006 SD/y; *P* = .002) in the CHARLS cohort (Table 3). A similar inverse association pattern was observed for eGFRcys in the HRS cohort (grip strength: β = 0.004 SD/y; 95% CI, 0.002-0.007 SD/y; *P* = .001; gait speed: β = 0.006 SD/y; 95% CI, 0.003-0.010 SD/y; *P* = .001) and the CHARLS cohort (gait speed: β = 0.021 SD/y; 95% CI, 0.009-0.034 SD/y; *P* = .001). Among participants in the CHARLS cohort, the associations of serum cystatin C (β = −0.016 SD/y; 95% CI, −0.027 to −0.006 SD/y; *P* = .003) and eGFRcys (β = 0.021 SD/y; 95% CI, 0.009-0.033 SD/y; *P* = .001) with decreases in gait speed remained evident after controlling for serum creatinine and eGFRcr, respectively. In the CHARLS cohort, each SD eGFRdiff increment was associated with slower decreases in both grip strength (β = 0.007 SD/y; 95% CI, 0.001-0.013 SD/y; *P* = .03) and gait speed (β = 0.017 SD/y; 95% CI, 0.005-0.028 SD/y; *P* = .005).

**Table 3.** Longitudinal Associations Between Kidney Function Measures and Rate of Change in Physical Function Among 2 Independent Cohorts Based on Inverse Probability–Weighted Samples

Kidney function measure	Rate of change in grip strength, SD/y		Rate of change in gait speed, SD/y	
	β (95% CI)^a^	*P* value	β (95% CI)^a^	*P* value
**HRS (n = 9114)**				
eGFRcys, mL/min/1.73 m^2^				
<60	−0.011 (−0.017 to −0.005)	.001	−0.021 (−0.030 to −0.011)	.001
60-89	−0.006 (−0.011 to −0.002)	.006	−0.012 (−0.020 to −0.004)	.005
≥90	0 [Reference]	NA	0 [Reference]	NA
Test for linear trend^b^	−0.005 (−0.008 to −0.003)	.001	−0.009 (−0.014 to −0.005)	.001
Continuous serum cystatin C per SD^c^	−0.006 (−0.008 to −0.003)	.001	−0.007 (−0.011 to −0.003)	.001
Continuous eGFRcys per SD^c^	0.004 (0.002 to 0.007)	.001	0.006 (0.003 to 0.010)	.001
**CHARLS (n = 6835)**				
Categories by eGFRcys, mL/min/1.73 m^2^				
<60	−0.018 (−0.037 to 0.002)	.07	−0.048 (−0.082 to −0.013)	.006
60-89	−0.000 (−0.015 to 0.014)	.95	−0.002 (−0.033 to 0.029)	.91
≥90	0 [Reference]	NA	0 [Reference]	NA
Test for linear trend^b^	−0.007 (−0.017 to 0.002)	.12	−0.025 (−0.042 to −0.008)	.003
Continuous serum cystatin C per SD^c^	−0.006 (−0.013 to 0.000)	.05	−0.017 (−0.027 to −0.006)	.002
Continuous eGFRcys per SD^c^	0.005 (−0.001 to 0.011)	.13	0.021 (0.009 to 0.034)	.001
Continuous serum creatinine per SD^c^	−0.001 (−0.007 to 0.006)	.83	0.006 (−0.004 to 0.017)	.24
Continuous eGFRcr per SD^c^	−0.002 (−0.008 to 0.005)	.58	0.007 (−0.004 to 0.019)	.21
Continuous cystatin C adjusted for creatinine per SD^c^	−0.006 (−0.013 to 0.001)	.07	−0.016 (−0.027 to −0.006)	.003
Continuous eGFRcys adjusted for eGFRcr per SD^c^	0.005 (−0.002 to 0.011)	.15	0.021 (0.009 to 0.033)	.001
eGFRdiff adjusted for eGFRcr per SD^c^	0.007 (0.001 to 0.013)	.03	0.017 (0.005 to 0.028)	.005

Abbreviations: CHARLS, China Health and Retirement Longitudinal Study; eGFRcr, estimated glomerular filtration rate using serum creatinine; eGFRcys, eGFR using serum cystatin C; eGFRdiff, difference between eGFRcys and eGFRcr (calculated as eGFRcys − eGFRcr); HRS, Health and Retirement Study; NA, not applicable.

^a^ β Coefficient was estimated using linear mixed models. Adjusted covariates include age, sex, race and ethnicity, living alone, educational level, annual household income, employment status, health insurance status, current smoking, alcohol consumption, physical activity, overweight status, physical disability, hypertension, diabetes, stroke, heart diseases, chronic lung diseases, and cancer.

^b^ Performed by treating eGFRcys categories as a numerical variable.

^c^ Estimated as the β coefficient for 1 SD increment in kidney measures.

### Sensitivity Analyses

A nonresponse analysis comparing baseline characteristics of included vs excluded participants was performed to evaluate potential selection bias. Compared with included participants, excluded participants (9335 from the HRS and 10 873 from the CHARLS) were generally older (HRS cohort: mean [SD] age, 69.7 [11.7] years vs 66.2 [10.1] years; CHARLS cohort: mean [SD] age, 58.6 [10.4] years vs 58.4 [9.8] years) and had more progressed frailty (HRS cohort: median FI score, 0.26 [IQR, 0.12-0.40] vs 0.11 [IQR, 0.05-0.16]; CHARLS cohort: median FI score, 0.13 [IQR, 0.06-0.28] vs 0.09 [IQR, 0.05-0.16]) (eTable 4 and eTable 5 in the Supplement).

The group-based trajectory modeling approach consistently identified 3 frailty trajectories in both cohorts, including increases in accelerated frailty, moderate frailty, and stable frailty (eFigure 2 in the Supplement). In the multinomial logistic regression analysis, we found a similar association pattern between cystatin C kidney function measures and the odds of increases in accelerated frailty trajectories (eg, serum cystatin C level per SD in HRS cohort: odds ratio [OR], 1.22; 95% CI, 1.12-1.33; *P* = .001; serum cystatin C level per SD in CHARLS cohort: OR, 1.22; 95% CI, 1.10-1.35; *P* = .001) and moderate frailty trajectories (eg, serum cystatin C level per SD in HRS cohort: OR, 1.16; 95% CI, 1.08-1.25; *P* = .001; serum cystatin C level per SD in CHARLS cohort: OR, 1.08; 95% CI, 1.01-1.16; *P* = .03) (eTable 6 in the Supplement). After conducting the primary analysis using the original unweighted samples, results were not substantially changed (eTable 7 and eTable 8 in the Supplement).

## Discussion

Based on 12-year follow-up data from the HRS and 7-year follow-up data from the CHARLS, this cohort study found that serum cystatin C level, eGFRcys, and eGFRdiff were associated with accelerated deficit-accumulation frailty trajectories and faster decreases in physical function measurements. In addition, adjustment for serum creatinine level and eGFRcr did not substantially change these findings. To our knowledge, this prospective study was the first to simultaneously evaluate the prospective associations of serum cystatin C, eGFRcys, and eGFRdiff with long-term deficit-accumulation frailty trajectories and physical function decline, with robust findings based on data from 2 population-based cohorts of community-dwelling older people without frailty at baseline.

Previous studies^29,30^ have explored associations between kidney function measures and frailty. In the Atherosclerosis Risk in Communities Study,^29^ researchers found that frailty was associated with reductions in eGFRcys. In another cohort study,^30^ researchers found that lower eGFRcys was associated with a higher risk of incident frailty, whereas eGFRcr was not. Despite similar findings, these studies generally focused on frailty status instead of frailty trajectories.

Our findings regarding the associations of cystatin C level and eGFRcys with physical function decline were consistent with those of previous studies,^31,32^ and we further found that the eGFRdiff was associated with subsequent physical function decline. In the Framingham Offspring Study,^31^ researchers found that individuals diagnosed with chronic kidney disease (using eGFRcys) experienced a faster decrease in gait speed in comparison with individuals without chronic kidney disease. Another study^32^ found that cystatin C level was consistently associated with faster decreases in gait speed and grip strength.

Our findings suggested that cystatin C level and eGFRcys could have clinical value beyond creatinine level and eGFRcr. One of the major superiorities of using cystatin C vs creatinine is the capability of estimating GFR while eliminating negative consequences from racial disparities.^7^ Our study applied the latest race-free equation to estimate eGFRcr, and we still only observed associations for cystatin C and eGFRcys. Hence, cystatin C itself could serve as a better marker than creatinine, not only for measuring kidney function but for assessing the risk markers of accelerated frailty and physical function decline. In addition, consistent with our hypothesis, we found that the higher positive eGFRdiff was consistently associated with a slower increase in frailty and a slower decrease in physical function. This finding was another notable implication of the potential value of eGFRdiff. Because the negative eGFRdiff could indicate potential sarcopenia, the possibility exists that the marker might have utility beyond kidney function.^8,12^ Despite the fact that conventional measures typically combine cystatin C and creatinine to derive more accurate eGFRs, we found that the relative difference in calculated GFRs also provided important information and could be used to identify those at high risk of experiencing accelerated frailty and physical function decline in the long term. Acknowledgment of such implications could enable clinicians to improve their practice, not only in kidney function monitoring but in risk identification of adverse health consequences among aging individuals.

### Strengths and Limitations

This study has several strengths. First, with up to 12 years of follow-up data, we were able to evaluate long-term dynamic frailty trajectories based on multiple repeated measurements. Second, we also incorporated objective measurements of physical function into the analysis, with multiple indices for evaluating the decline in physical function. Third, our findings were robust, with similar results observed in various sensitivity analyses. Fourth, our study population was large and nationally representative of community-dwelling older adults.

The study also has limitations. First, criterion-standard GFR was not measured, and only eGFR was used for analysis. Second, we excluded a large number of participants, which may have produced selection bias. Although an inverse probability weighting approach was applied to handle this issue, the possibility of selection bias could not be eliminated. Third, the 2 cohorts we included had many different characteristics. The significant heterogeneity between the 2 cohorts prevented us from conducting a pooled analysis. In addition, only dried blood samples were used to measure kidney function in the HRS, which prevented the generalization of findings and might explain the different findings regarding physical function decline. The HRS also did not measure creatinine level, decreasing the validity of findings regarding eGFRdiff. Fourth, the total number of deficits included for FI calculation was insufficient and could lead to inaccurate or unstable findings. According to previous studies,^19,33,34^ an FI could have been sufficiently robust or accurate for estimating adverse outcomes when the total number of deficits reached 30 to 40. Fifth, because most deficits included in the FI calculation were self-reported, the potential for information bias could not be eliminated. Sixth, due to the nature of observational studies, we could not eliminate the possibility of residual confounding, which impedes further steps toward conclusively establishing causal relationships.^35^

## Conclusions

This cohort study found that serum cystatin C, eGFRcys, and eGFRdiff were associated with long-term accelerated frailty trajectories and physical function decline among community-dwelling older people without frailty. Monitoring of kidney function using cystatin C could have clinical utility for identifying the risk of accelerated frailty progression.

## Notes

Supplement. **eMethods.** Frailty Assessment and Statistical Analysis **eTable 1.** Components of Constructed 29-Item Frailty Index **eTable 2.** Descriptive Statistics of Outcome Measurements at Different Waves in the HRS Cohort **eTable 3.** Descriptive Statistics of Outcome Measurements at Different Waves in the CHARLS Cohort **eTable 4.** Comparison of Baseline Characteristics of HRS Participants Included in vs Excluded From Analysis **eTable 5.** Comparison of Baseline Characteristics of CHARLS Participants Included in vs Excluded From Analysis **eTable 6.** Association Between Kidney Function Measures and Frailty Trajectories Identified Using the GBTM Approach in 2 Independent Cohorts Based on the Original Unweighted Samples **eTable 7.** Longitudinal Associations Between Kidney Function Measures and Frailty Trajectories in 2 Independent Cohorts Based on the Original Unweighted Samples **eTable 8.** Longitudinal Associations Between Kidney Function Measures and Rate of Change in Physical Function in 2 Independent Cohorts Based on the Original Unweighted Samples **eFigure 1.** Participant Selection Diagram **eFigure 2.** Identified Dynamic Frailty Trajectories in 2 Independent Cohorts Using the GBTM Approach
eReferences
