# Supplementary material for: Association of Cystatin C Kidney Function Measures With Long-term Deficit-Accumulation Frailty Trajectories and Physical Function Decline
Source: JAMA Netw Open. 2022 Sep 30;5(9):e2234208. doi: 10.1001/jamanetworkopen.2022.34208 (PMC9526088; doi:10.1001/jamanetworkopen.2022.34208)
Supplement: Supplement. — eMethods. Frailty Assessment and Statistical Analysis eTable 1. Components of Constructed 29-Item Frailty Index eTable 2. Descriptive Statistics of Outcome Measurements at Different Waves in the HRS Cohort eTable 3. Descriptive Statistics of Outcome Measurements at Different Waves in the CHARLS Cohort eTable 4. Comparison of Baseline Characteristics of HRS Participants Included in vs Excluded From Analysis eTable 5. Comparison of Baseline Characteristics of CHARLS Participants Included in vs Excluded From Analysis eTable 6. Association Between Kidney Function Measures and Frailty Trajectories Identified Using the GBTM Approach in 2 Independent Cohorts Based on the Original Unweighted Samples eTable 7. Longitudinal Associations Between Kidney Function Measures and Frailty Trajectories in 2 Independent Cohorts Based on the Original Unweighted Samples eTable 8. Longitudinal Associations Between Kidney Function Measures and Rate of Change in Physical Function in 2 Independent Cohorts Based on the Original Unweighted Samples eFigure 1. Participant Selection Diagram eFigure 2. Identified Dynamic Frailty Trajectories in 2 Independent Cohorts Using the GBTM Approach eReferences [file jamanetwopen-e2234208-s001.pdf]

Supplementary Online Content

Li C, Ma Y, Yang C, Hua R, Xie W, Zhang L. Association of cystatin C kidney function measures with long-term deficit-accumulation frailty trajectories and physical function decline. *JAMA Netw Open*. 2022;5(9):e2234208. doi:10.1001/jamanetworkopen.2022.34208

eMethods. Frailty Assessment and Statistical Analysis

eTable 1. Components of Constructed 29-Item Frailty Index

eTable 2. Descriptive Statistics of Outcome Measurements at Different Waves in the HRS Cohort

eTable 3. Descriptive Statistics of Outcome Measurements at Different Waves in the CHARLS Cohort

eTable 4. Comparison of Baseline Characteristics of HRS Participants Included in vs Excluded From Analysis

eTable 5. Comparison of Baseline Characteristics of CHARLS Participants Included in vs Excluded From Analysis

eTable 6. Association Between Kidney Function Measures and Frailty Trajectories Identified Using the GBTM Approach in 2 Independent Cohorts Based on the Original Unweighted Samples

eTable 7. Longitudinal Associations Between Kidney Function Measures and Frailty Trajectories in 2 Independent Cohorts Based on the Original Unweighted Samples

eTable 8. Longitudinal Associations Between Kidney Function Measures and Rate of Change in Physical Function in 2 Independent Cohorts Based on the Original Unweighted Samples

eFigure 1. Participant Selection Diagram

eFigure 2. Identified Dynamic Frailty Trajectories in 2 Independent Cohorts Using the GBTM Approach

eReferences

This supplementary material has been provided by the authors to give readers additional information about their work.

© 2022 Li C et al. *JAMA Network Open*.

eMethods. Frailty Assessment and Statistical Analysis

1. Frailty assessment

The 29 items used to construct the FI included functional limitations (based on self-reported difficulties in activities of daily living, instrumental activities of daily living, and other activities), self-reported health status and alterations, components of depressive symptoms (based on 8-item version of the Center for Epidemiologic Studies Depression Scale), medical conditions (based on self-reported diagnosis by physicians), and cognitive status (based on a combination of external physician diagnosis and cognition score). These items came from self-reported information by study participants and objective measurements. The functional limitations were derived from the Activities of Daily Living (ADLs) and Instrumental Activities of Daily Living (IADLs), as well as other self-reported subjective activities limitations. Self-reported health status and alterations were derived using the self-rating of participants' overall health status. Components of depressive symptoms were extracted from 8-item version of the Center for Epidemiologic Studies Depression Scale, which included 8 questions concerning indicators of participants' feelings much of the time over the week prior to the interview, with each question consisting of 2 response categories (yes or no). The medical conditions were extracted from self-reported physician diagnosis of relevant diseases, with confirmation procedure applied at each wave. Such procedure was applied to help participants confirm whether they had been diagnosed of the disease at previous wave. The cognitive status was measured based on a combination of self-reported diagnosis and cognition score. We defined dementia cases using either a self-reported physician diagnosis or an alternative approach based on cognition scores. For the HRS, we used a cognition summary score from 0 to 27, with a cutoff point of 6 or less defined as dementia and 7 to 11 defined as cognitive impairment.[1] The cognition summary score comprised the domains of memory and executive function.[1] For the CHARLS, we defined dementia as a coexistence of cognitive and functional impairment.[2] Cognitive impairment was defined as a score that was 1.5 SD below the mean of the population stratified by educational background.[2] Functional impairment was defined as difficulty in performing one or more activities of daily living, including bathing, eating, dressing, getting in/out of bed, and walking across a room. These approaches had been validated.[1–3]

2. Statistical analysis

Based on the 12-year repeated measurements of FI in the HRS and 7-year data in the CHARLS, we alternatively used the GBTM approach to evaluate long-term frailty trajectories. The GBTM approach assumed that the total population was consisted of several subpopulations with different longitudinal trajectories, and used maximum likelihood estimation to identify participants sharing similar trajectories.[4] The trajectory group of the highest probability was determined for each participant, which was then included in further multivariate analysis. The GBTM approach can fit non-monotonic trajectories and support multiple trajectory shapes including linear, quadratic and cubic. It also allows specification of number of trajectory groups before fitting the model.

After determining that modeling 3 trajectory groups was appropriate for frailty trajectories modeling, we further evaluated different trajectory shapes for each trajectory group by testing the null hypothesis that the shape parameter for the group equals zero. We also used graphics of trajectory group means to help determine which shape best fit each trajectory group. After the procedure, we determined that the best 3-group trajectory model consisted of 3 quadratic trajectories. Then the estimated trajectory groups membership was included as the dependent variable for further multivariate analysis. We analyzed the associations between baseline kidney function metrics and identified frailty trajectories using binary logistic and multinomial logistic regression models, respectively.

© 2022 Li C et al. *JAMA Network Open*.

eTable 1. Components of Constructed 29-Item Frailty Index

Item number [a]	Item definition [b]	Scoring [c]
1	Self-reported difficulties in bathing because of a physical, mental, emotional or memory problem.	Yes=1.00; No=0.00
2	Self-reported difficulties in dressing because of a physical, mental, emotional or memory problem.	Yes=1.00; No=0.00
3	Self-reported difficulties in getting in/out of bed because of a physical, mental, emotional or memory problem.	Yes=1.00; No=0.00
4	Self-reported difficulties in walking around the house because of a physical, mental, emotional or memory problem.	Yes=1.00; No=0.00
5	Self-reported difficulties in eating because of a physical, mental, emotional or memory problem.	Yes=1.00; No=0.00
6	Self-reported difficulties in finishing daily activities of using the toilet because of a physical, mental, emotional or memory problem.	Yes=1.00; No=0.00
7	Total number (0-5) of self-reported difficulties in finishing daily activities including bathing, dressing, eating, getting in/out of bed, walking around the house.	Any difficulties=1.00; No difficulties=0.00
8	Self-reported difficulties in shopping because of a physical, mental, emotional or memory problem.	Yes=1.00; No=0.00
9	Self-reported difficulties in preparing hot meal because of a physical, mental, emotional or memory problem.	Yes=1.00; No=0.00
10	Self-reported difficulties in taking prescribed medications because of a physical, mental, emotional or memory problem.	Yes=1.00; No=0.00
11	Self-reported difficulties in managing money because of a	Yes=1.00; No=0.00

© 2022 Li C et al. *JAMA Network Open.*

Item number [a]	Item definition [b]	Scoring [c]
	physical, mental, emotional or memory problem.	
12	Self-reported difficulties in getting up from a chair because of a physical, mental, emotional or memory problem.	Yes=1.00; No=0.00
13	Self-reported difficulties in climbing several flights of stairs because of a physical, mental, emotional or memory problem.	Yes=1.00; No=0.00
14	Self-reported difficulties in lifting or carrying weights over 10 pounds because of a physical, mental, emotional or memory problem.	Yes=1.00; No=0.00
15	Self-reported difficulties in walking one block because of a physical, mental, emotional or memory problem.	Yes=1.00; No=0.00
16	Self-reported rating of health status.	Poor=1.00; Fair=0.75; Good=0.50; Very Good=0.25; Excellent=0.00
17	Change in self-reported rating of health status.	Worse=1.00; Better/Same=0.00
18	Feeling that everything is an effort much of time.	Yes=1.00; No=0.00
19	Feeling depressed much of time.	Yes=1.00; No=0.00
20	Feeling happy much of time.	No=1.00; Yes=0.00
21	Feeling lonely much of time.	Yes=1.00; No=0.00
22	Feeling that could not get going much of time.	Yes=1.00; No=0.00
23	Self-reported diagnosis of hypertension by physician.	Yes=1.00; No=0.00
24	Self-reported diagnosis of stroke by physician.	Yes=1.00; No=0.00
25	Self-reported diagnosis of cancer by physician.	Yes=1.00; No=0.00
26	Self-reported diagnosis of diabetes by physician.	Yes=1.00; No=0.00
27	Self-reported diagnosis of arthritis by physician.	Yes=1.00; No=0.00
28	Self-reported diagnosis of chronic lung disease by physician.	Yes=1.00; No=0.00
29	Cognitive status, based on combination of self-reported diagnosis, and cognition score.	Dementia=1.00; Cognitive impairment but not demented=0.50; Cognitive healthy=0.00

© 2022 Li C et al. *JAMA Network Open.*

Item number [a]	Item definition [b]	Scoring [c]

[a] An unified 29-item frailty index was utilized across different waves, identical in both the HRS and the CHARLS.

[b] Item definition was based on self-reported information or objective measurements or both.

[c] The frailty index was calculated as the sum of scoring divided by the total number of items.

© 2022 Li C et al. *JAMA Network Open*.

eTable 2. Descriptive Statistics of Outcome Measurements at Different Waves in the HRS Cohort

**Waves**	**Frailty index**		**Grip strength (kg)**	**Gait speed (cm/s)**
	**mean (SD)**	**median (IQR)**	**mean (SD)**	**mean (SD)**
Wave 8 (year 2006)	0.11 (0.07)	0.11 (0.05-0.16)	32.79 (11.03)	84.99 (23.87)
Wave 9 (year 2008)	0.13 (0.10)	0.12 (0.07-0.18)	NA	NA
Wave 10 (year 2010)	0.15 (0.12)	0.12 (0.08-0.20)	30.78 (10.53)	84.21 (23.94)
Wave 11 (year 2012)	0.16 (0.13)	0.13 (0.08-0.22)	NA	NA
Wave 12 (year 2014)	0.18 (0.14)	0.15 (0.08-0.22)	28.94 (10.54)	80.70 (23.24)
Wave 13 (year 2016)	0.18 (0.14)	0.16 (0.09-0.23)	NA	NA
Wave 14 (year 2018)	0.16 (0.13)	0.12 (0.08-0.20)	28.56 (12.70)	81.00 (23.99)

Abbreviations: HRS, Health and Retirement Study; SD, standard deviation; IQR, interquartile range; NA, not applicable.

© 2022 Li C et al. *JAMA Network Open*.

eTable 3. Descriptive Statistics of Outcome Measurements at Different Waves in the CHARLS Cohort

Waves	Frailty index		Grip strength (kg)	Gait speed (cm/s)
	mean (SD)	median (IQR)	mean (SD)	mean (SD)
Wave 1 (year 2011)	0.10 (0.07)	0.09 (0.05-0.16)	32.36 (10.45)	64.56 (21.01)
Wave 2 (year 2013)	0.15 (0.11)	0.13 (0.08-0.20)	31.30 (10.91)	69.32 (19.96)
Wave 3 (year 2015)	0.17 (0.13)	0.13 (0.08-0.22)	29.55 (10.30)	78.03 (21.66)
Wave 4 (year 2018)	0.18 (0.14)	0.14 (0.08-0.24)	NA	NA

Abbreviations: CHARLS, China Health and Retirement Longitudinal Study; SD, standard deviation; IQR, interquartile range; NA, not applicable.

© 2022 Li C et al. *JAMA Network Open*.

eTable 4. Comparison of Baseline Characteristics of HRS Participants Included in vs Excluded From Analysis

Characteristics	No. (%)		
	Excluded n=9355	Included n=9114	*P* [a]
Men	3716 (39.72)	3870 (42.46)	.001
Age, mean (SD), y	69.74±11.72	66.19±10.11	.001
Follow-up duration, median (IQR), y	8.00 (2.00-12.00)	12.00 (8.00-12.00)	.001
White	7181 (76.76)	7755 (85.09)	.001
Living alone	3959 (42.32)	2458 (26.97)	.001
Education background			
Less than high school	2784 (29.76)	1372 (15.05)	.001
High school or equivalent	3203 (34.24)	3316 (36.38)	
College and above	3368 (36.00)	4426 (48.56)	
Family annual income thirds			
T1	4076 (43.57)	2085 (22.88)	.001
T2	2913 (31.14)	3238 (35.53)	
T3	2366 (25.29)	3791 (41.60)	
Employment status			
Unemployed	1504 (16.08)	843 (9.25)	.001
Employed	7851 (83.92)	8271 (90.75)	
Medical insurance coverage			
Uninsured	530 (5.67)	507 (5.56)	.001
Public insurance	3676 (39.29)	2237 (24.54)	
Private insurance	5149 (55.04)	6370 (69.89)	
Current smoking	1390 (14.86)	1141 (12.52)	.001
Alcohol consumption	1226 (13.11)	1834 (20.12)	.001
Physical activity	5432 (58.07)	7487 (82.15)	.001
Physical disability	3193 (34.13)	193 (2.12)	.001
Overweight	4345 (46.45)	6608 (72.50)	.001
Frailty index, median (IQR)	0.26 (0.12-0.40)	0.11 (0.05-0.16)	.001
Grip strength, mean (SD), kg	28.87±11.02	32.79±11.03	.001
Gait speed, mean (SD), cm/s	70.56±26.71	84.99±23.87	.001
Hypertension	6591 (70.45)	5682 (62.34)	.001
Diabetes	2513 (26.86)	1470 (16.13)	.001
Stroke	1280 (13.68)	344 (3.77)	.001
Heart diseases	2844 (30.40)	1572 (17.25)	.001
Chronic lung diseases	1213 (12.97)	427 (4.69)	.001
Cancer	1535 (16.41)	1040 (11.41)	.001

Abbreviations: HRS, Health and Retirement Study; SD, standard deviation; IQR, interquartile range.
[a] P value reported for differences between groups using t test, chi-square test, or Wilcoxon rank test.

© 2022 Li C et al. *JAMA Network Open*.

eTable 5. Comparison of Baseline Characteristics of CHARLS Participants Included in vs Excluded From Analysis

Characteristics	No. (%)		
	**Excluded n=10873**	**Included n=6835**	***P*** [a]
Men	5120 (47.09)	3358 (49.13)	.01
Age, mean (SD), y	58.55±10.41	58.41±9.79	.37
Follow-up duration, median (IQR), y	7.00 (4.00-7.00)	7.00 (7.00-7.00)	.001
Living alone	1501 (13.80)	759 (11.10)	.001
Education background			
Less than high school	9525 (87.60)	6036 (88.31)	.22
High school or equivalent	1117 (10.27)	676 (9.89)	
College and above	231 (2.12)	123 (1.80)	
Family annual income thirds			
T1	3722 (34.23)	2244 (32.83)	.15
T2	3583 (32.95)	2288 (33.47)	
T3	3568 (32.82)	2303 (33.69)	
Employment status			
Unemployed	603 (5.55)	260 (3.80)	.001
Employed	10270 (94.45)	6575 (96.20)	
Medical insurance coverage			
Uninsured	1040 (9.56)	435 (6.36)	.001
Public insurance	9581 (88.12)	6214 (90.91)	
Private insurance	252 (2.32)	186 (2.72)	
Current smoking	2817 (25.91)	2054 (30.05)	.001
Alcohol consumption	1298 (11.94)	1005 (14.70)	.001
Physical activity	2488 (22.88)	1924 (28.15)	.001
Physical disability	2386 (21.94)	283 (4.14)	.001
Overweight	3915 (36.01)	2750 (40.23)	.001
Frailty index, median (IQR)	0.13 (0.06-0.28)	0.09 (0.05-0.16)	.001
Grip strength, mean (SD), kg	30.83±11.06	32.36±10.45	.001
Gait speed, mean (SD), cm/s	59.63±21.79	64.56±21.01	.001
Hypertension	4492 (41.31)	2667 (39.02)	.003
Diabetes	1461 (13.44)	1020 (14.92)	.006
Stroke	398 (3.66)	88 (1.29)	.001
Cardiovascular disease	1393 (12.81)	737 (10.78)	.001
Chronic lung disease	1156 (10.63)	548 (8.02)	.001
Cancer	121 (1.11)	46 (0.67)	.004

Abbreviations: CHARLS, China Health and Retirement Longitudinal Study; SD, standard deviation; IQR, interquartile range.

[a] P value reported for differences between groups using t test, chi-square test, or Wilcoxon rank test.

© 2022 Li C et al. *JAMA Network Open*.

eTable 6. Association Between Kidney Function Measures and Frailty Trajectories Identified Using the GBTM Approach in 2 Independent Cohorts Based on the Original Unweighted Samples

	**Binary logistic regression [a]**		**Multinomial logistic regression [b]**			
	**Accelerated frailty increase**		**Accelerated frailty increase**		**Moderate frailty increase**	
**Kidney function metrics**	**OR (95% CI)**	***P***	**OR (95% CI)**	***P***	**OR (95% CI)**	***P***
**HRS (n=9114)**						
Categories by eGFRCys						
≥ 90 mL/min/1.73m^2	1 [Reference]	NA	1 [Reference]	NA	1 [Reference]	NA
60-89 mL/min/1.73m^2	1.06 (0.85 - 1.32)	.60	1.07 (0.85 - 1.37)	.14	1.08 (0.94 - 1.24)	.43
< 60 mL/min/1.73m^2	1.29 (1.03 - 1.63)	.03	1.52 (1.17 - 1.97)	.001	1.27 (1.07 - 1.50)	.005
Test for linear trend [c]	1.15 (1.03 - 1.29)	.01	1.25 (1.10 - 1.43)	.001	1.12 (1.03 - 1.22)	.007
Continuous serum cystatin C, per SD [d]	1.07 (1.01 - 1.14)	.03	1.22 (1.12 - 1.33)	.001	1.16 (1.08 - 1.25)	.001
Continuous eGFRCys, per SD	0.89 (0.81 - 0.96)	.005	0.81 (0.73 - 0.89)	.001	0.88 (0.83 - 0.94)	.001
**CHARLS (n=6835)**						
Categories by eGFRCys						
≥ 90 mL/min/1.73m^2	1 [Reference]	NA	1 [Reference]	NA	1 [Reference]	NA
60-89 mL/min/1.73m^2	1.03 (0.78 - 1.35)	.83	1.06 (0.80 - 1.41)	.16	1.08 (0.93 - 1.26)	.58
< 60 mL/min/1.73m^2	1.49 (1.07 - 2.07)	.02	1.53 (1.08 - 2.17)	.005	1.09 (0.88 - 1.36)	.60
Test for linear trend	1.23 (1.04 - 1.46)	.01	1.26 (1.05 - 1.50)	.01	1.05 (0.94 - 1.17)	.37
Continuous serum cystatin C, per SD	1.18 (1.07 - 1.29)	.001	1.22 (1.10 - 1.35)	.001	1.08 (1.01 - 1.16)	.03
Continuous eGFRCys, per SD	0.83 (0.73 - 0.94)	.003	0.80 (0.70 - 0.91)	.001	0.92 (0.85 - 0.99)	.04
Continuous serum creatinine, per SD	0.91 (0.81 - 1.02)	.12	0.92 (0.81 - 1.03)	.16	0.99 (0.92 - 1.07)	.85
Continuous eGFRCr, per SD	1.09 (0.97 - 1.23)	.14	1.08 (0.95 - 1.22)	.25	0.99 (0.92 - 1.07)	.74
Continuous cystatin C adjusted for creatinine, per SD	1.33 (1.19 - 1.49)	.001	1.40 (1.24 - 1.58)	.001	1.12 (1.03 - 1.21)	.008
Continuous eGFRCys adjusted for eGFRCr, per SD	0.75 (0.66 - 0.87)	.001	0.73 (0.63 - 0.84)	.001	0.91 (0.83 - 0.99)	.03
eGFRdiff adjusted for eGFRCr, per SD	0.79 (0.71 - 0.89)	.001	0.77 (0.68 - 0.87)	.001	0.93 (0.86 - 0.99)	.03

Abbreviations: CHARLS, China Health and Retirement Longitudinal Study; HRS, Health and Retirement Study; SD, standard deviation; CI, confidence interval; OR, odds ratio; eGFRCys, estimated glomerular filtration rate using serum cystatin C; eGFRCr, estimated glomerular filtration rate using serum creatinine; eGFRdiff, difference between eGFRCys and eGFRCr (calculated as eGFRCys - eGFRCr); NA, not applicable.
[a] OR was estimated using binary logistic regression models, with the logit link function. Accelerated frailty progression trajectory was the outcome, while the moderate and stable frailty progression trajectories were

© 2022 Li C et al. *JAMA Network Open.*

combined as the reference category for modelling.

[b] OR was estimated using multinomial logistic regression models, with the generalized logit link function. Accelerated frailty progression trajectory and moderate frailty trajectory were outcomes, while the stable frailty progression trajectory was the reference category for modeling.

Adjusted covariates included age, sex, ethnicity, living arrangement, education background, family annual income, employment status, medical insurance coverage, current smoking, alcohol consumption, physical activity, overweight status, physical disability, baseline frailty index, hypertension, diabetes, stroke, heart diseases, chronic lung diseases, and cancer.

[c] Performed by treating eGFRCys categories as a numerical variable.

[d] Estimated as the beta coefficient for 1 SD increment in kidney metrics.

© 2022 Li C et al. *JAMA Network Open*.

eTable 7. Longitudinal Associations Between Kidney Function Measures and Frailty Trajectories in 2 Independent Cohorts Based on the Original Unweighted Samples

Kidney function metrics	**Rate of change in frailty index (SD/year)**	
	**β (95% CI)** [a]	***P***
**HRS (n=9114)**		
Categories by eGFRCys		
≥ 90 mL/min/1.73m^2	0 [Reference]	NA
60-89 mL/min/1.73m^2	0.041 (0.031 to 0.052)	.001
< 60 mL/min/1.73m^2	0.136 (0.124 to 0.148)	.001
Test for linear trend [b]	0.066 (0.060 to 0.072)	.001
Continuous serum cystatin C, per SD [c]	0.048 (0.043 to 0.053)	.001
Continuous eGFRCys, per SD	-0.055 (-0.059 to -0.050)	.001
**CHARLS (n=6835)**		
Categories by eGFRCys		
≥ 90 mL/min/1.73m^2	0 [Reference]	NA
60-89 mL/min/1.73m^2	0.031 (0.012 to 0.050)	.001
< 60 mL/min/1.73m^2	0.167 (0.142 to 0.193)	.001
Test for linear trend	0.074 (0.062 to 0.087)	.001
Continuous serum cystatin C, per SD	0.053 (0.044 to 0.062)	.001
Continuous eGFRCys, per SD	-0.057 (-0.066 to -0.049)	.001
Continuous serum creatinine, per SD	0.004 (-0.005 to 0.013)	.40
Continuous eGFRCr, per SD	-0.048 (-0.057 to -0.039)	.001
Continuous cystatin C adjusted for creatinine, per SD	0.053 (0.044 to 0.062)	.001
Continuous eGFRCys adjusted for eGFRCr, per SD	-0.057 (-0.066 to -0.048)	.001
eGFRdiff adjusted for eGFRCr, per SD	-0.029 (-0.038 to -0.020)	.001

Abbreviations: CHARLS, China Health and Retirement Longitudinal Study; HRS, Health and Retirement Study; SD, standard deviation; CI, confidence interval; eGFRCys, estimated glomerular filtration rate using serum cystatin C; eGFRCr, estimated glomerular filtration rate using serum creatinine; eGFRdiff, difference between eGFRCys and eGFRCr (calculated as eGFRCys - eGFRCr); NA, not applicable.

[a] Beta coefficient was estimated using linear mixed models, positive value represents accelerated frailty. Adjusted covariates included age, sex, ethnicity, living arrangement, education background, family annual income, employment status, medical insurance coverage, current smoking, alcohol consumption, physical activity, overweight status, physical disability, hypertension, diabetes, stroke, heart diseases, chronic lung diseases, and cancer.

eGFRCys: estimated glomerular filtration rate using serum cystatin C; eGFRCr: estimated glomerular filtration rate using serum creatinine; eGFRdiff: difference between eGFRCys and eGFRCr, calculated as eGFRCys-eGFRCr.

[b] Performed by treating eGFRCys categories as a numerical variable.

[c] Estimated as the beta coefficient for 1 SD increment in kidney metrics.

© 2022 Li C et al. *JAMA Network Open*.

eTable 8. Longitudinal Associations Between Kidney Function Measures and Rate of Change in Physical Function in 2 Independent Cohorts Based on the Original Unweighted Samples

Kidney function metrics	Rate of change in grip strength (SD/year)		Rate of change in gait speed (SD/year)	
	β (95% CI) [a]	*P*	β (95% CI) [a]	*P*
**HRS (n=9114)**				
Categories by eGFRCys				
≥ 90 mL/min/1.73m^2	0 [Reference]	NA	0 [Reference]	NA
60-89 mL/min/1.73m^2	-0.007 (-0.011 to -0.002)	.004	-0.010 (-0.018 to -0.002)	.02
< 60 mL/min/1.73m^2	-0.010 (-0.015 to -0.004)	.001	-0.020 (-0.030 to -0.011)	.001
Test for linear trend [b]	-0.005 (-0.008 to -0.002)	.001	-0.009 (-0.014 to -0.005)	.001
Continuous serum cystatin C, per SD [c]	-0.005 (-0.008 to -0.003)	.001	-0.008 (-0.012 to -0.004)	.001
Continuous eGFRCys, per SD	0.004 (0.002 to 0.006)	.001	0.006 (0.003 to 0.010)	.001
**CHARLS (n=6835)**				
Categories by eGFRCys				
≥ 90 mL/min/1.73m^2	0 [Reference]	NA	0 [Reference]	NA
60-89 mL/min/1.73m^2	-0.004 (-0.018 to 0.011)	.62	-0.002 (-0.034 to 0.030)	.91
< 60 mL/min/1.73m^2	-0.023 (-0.042 to -0.004)	.02	-0.048 (-0.083 to -0.014)	.006
Test for linear trend	-0.010 (-0.019 to -0.001)	.03	-0.026 (-0.043 to -0.009)	.003
Continuous serum cystatin C, per SD	-0.008 (-0.015 to -0.002)	.01	-0.017 (-0.028 to -0.007)	.001
Continuous eGFRCys, per SD	0.007 (0.001 to 0.014)	.03	0.023 (0.010 to 0.035)	.001
Continuous serum creatinine, per SD	-0.001 (-0.008 to 0.005)	.68	0.006 (-0.005 to 0.017)	.27
Continuous eGFRCr, per SD	-0.000 (-0.007 to 0.006)	.89	0.009 (-0.003 to 0.021)	.15
Continuous cystatin C adjusted for creatinine, per SD	-0.008 (-0.014 to -0.002)	.01	-0.017 (-0.028 to -0.006)	.002
Continuous eGFRCys adjusted for eGFRCr, per SD	0.007 (0.001 to 0.013)	.03	0.022 (0.010 to 0.034)	.001
eGFRdiff adjusted for eGFRCr, per SD	0.009 (0.003 to 0.015)	.006	0.017 (0.005 to 0.029)	.005

Abbreviations: CHARLS, China Health and Retirement Longitudinal Study; HRS, Health and Retirement Study; SD, standard deviation; CI, confidence interval; eGFRCys, estimated glomerular filtration rate using serum cystatin C; eGFRCr, estimated glomerular filtration rate using serum creatinine; eGFRdiff, difference between eGFRCys and eGFRCr (calculated as eGFRCys - eGFRCr); NA, not applicable.

[a] Beta coefficient was estimated using linear mixed models. Adjusted covariates included age, sex, ethnicity, living arrangement, education background, family annual income, employment status, medical insurance coverage, current smoking, alcohol consumption, physical activity, overweight status, physical disability, hypertension, diabetes, stroke, heart diseases, chronic lung diseases, and cancer.

eGFRCys: estimated glomerular filtration rate using serum cystatin C; eGFRCr: estimated glomerular

© 2022 Li C et al. *JAMA Network Open*.

filtration rate using serum creatinine; eGFRdiff: difference between eGFRCys and eGFRCr, calculated as eGFRCys-eGFRCr.

[b] Performed by treating eGFRCys categories as a numerical variable.

[c] Estimated as the beta coefficient for 1 SD increment in kidney metrics.

© 2022 Li C et al. *JAMA Network Open*.

eFigure 1. Participant Selection Diagram

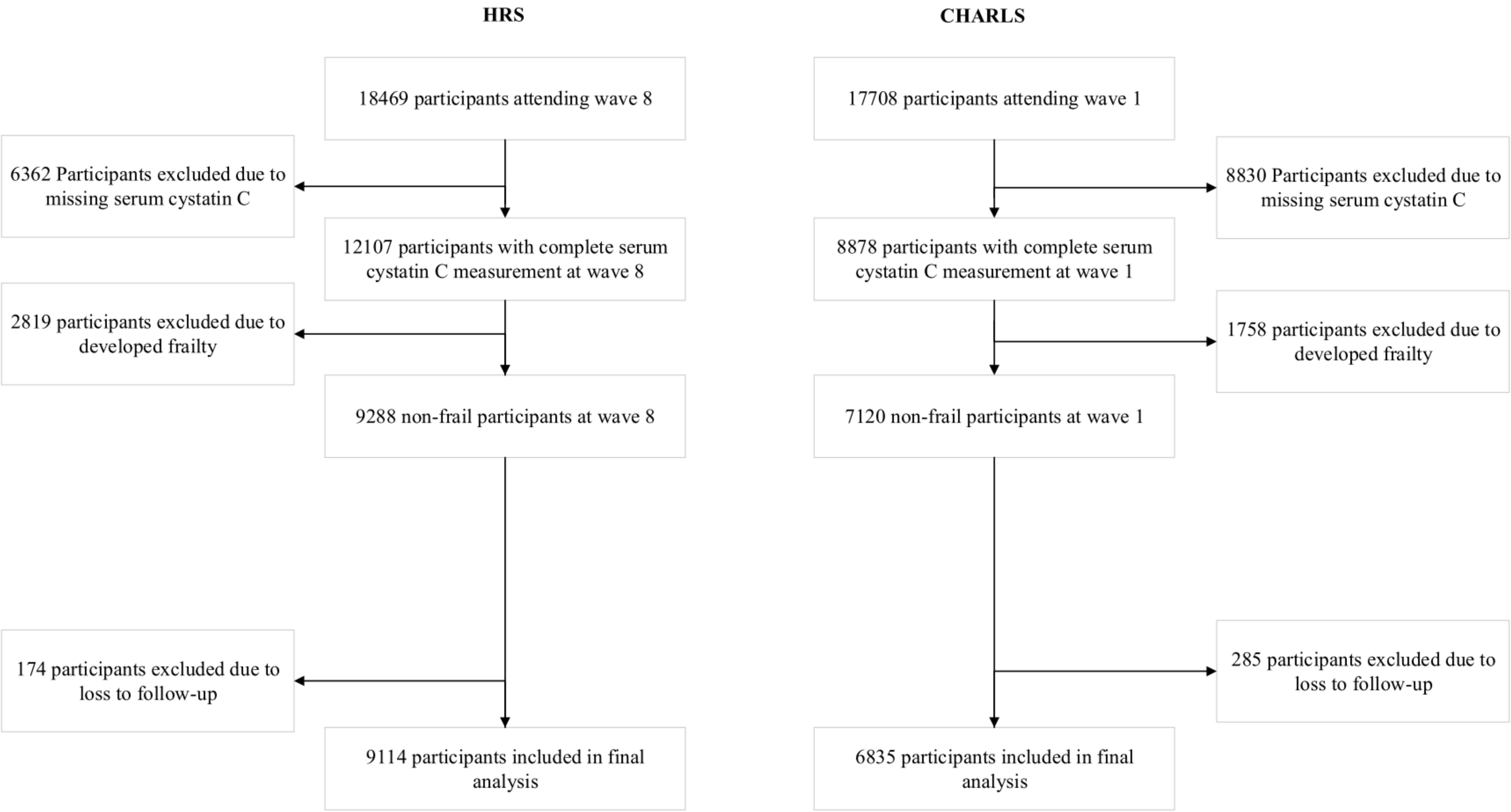

© 2022 Li C et al. *JAMA Network Open*.

eFigure 2. Identified Dynamic Frailty Trajectories in 2 Independent Cohorts Using the GBTM Approach

Panel A: HRS cohort; Panel B: CHARLS cohort.

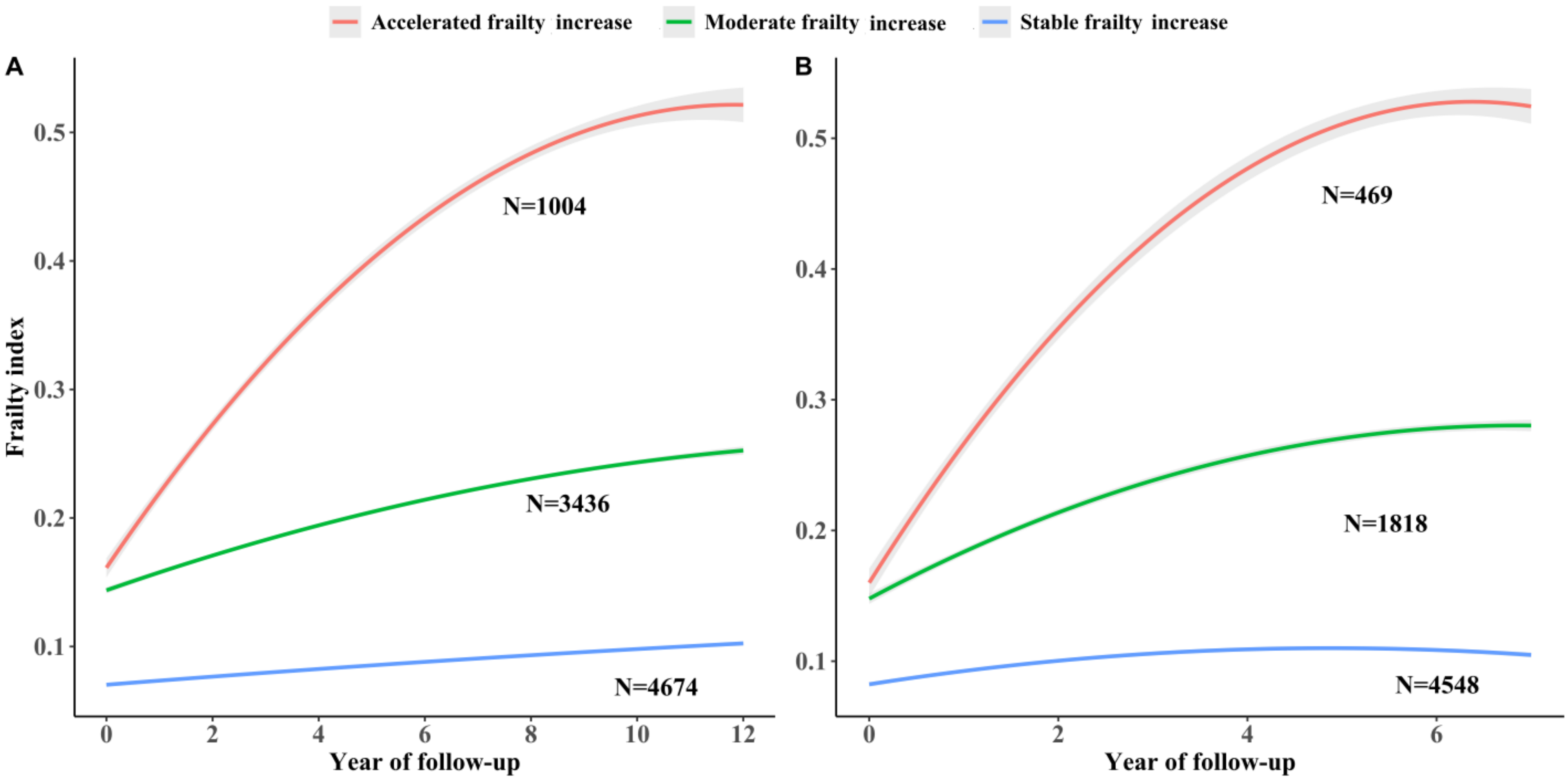

© 2022 Li C et al. *JAMA Network Open*.

eReferences

1. Crimmins EM, Kim JK, Langa KM, Weir DR. Assessment of cognition using surveys and neuropsychological assessment: the Health and Retirement Study and the Aging, Demographics, and Memory Study. *J Gerontol B Psychol Sci Soc Sci*. 2011;66 Suppl 1:i162-71. doi:10.1093/geronb/gbr048
2. Ahmadi-Abhari S, Guzman-Castillo M, Bandosz P, et al. Temporal trend in dementia incidence since 2002 and projections for prevalence in England and Wales to 2040: modelling study. *BMJ*. 2017;358:j2856. doi:10.1136/bmj.j2856
3. Grasset L, Glymour MM, Yaffe K, et al. Association of traumatic brain injury with dementia and memory decline in older adults in the United States. *Alzheimers Dement*. 2020;16:853-861. doi:10.1002/alz.12080
4. Jones BL, Nagin DS. Advances in Group-Based Trajectory Modeling and an SAS Procedure for Estimating Them. *Sociol Methods Res*. 2007;35:542-571. doi:10.1177/0049124106292364

© 2022 Li C et al. *JAMA Network Open.*
